# Supplementary material for: Inadequate foundational decoding skills constrain global literacy goals for pupils in low- and middle-income countries
Source: Nat Hum Behav. 2024 Nov 8;9(1):74–83. doi: 10.1038/s41562-024-02028-x (PMC11774748; doi:10.1038/s41562-024-02028-x)
Supplement: Supplementary file 1 — Supplementary Figs. 1–5, Tables 1–7 and Appendix. [file 41562_2024_2028_MOESM1_ESM.pdf]

nature human behaviour

Supplementary information https://doi.org/10.1038/s41562-024-02028-x

Inadequate foundational decoding skills constrain global literacy goals for pupils in low- and middle-income countries

In the format provided by the authors and unedited

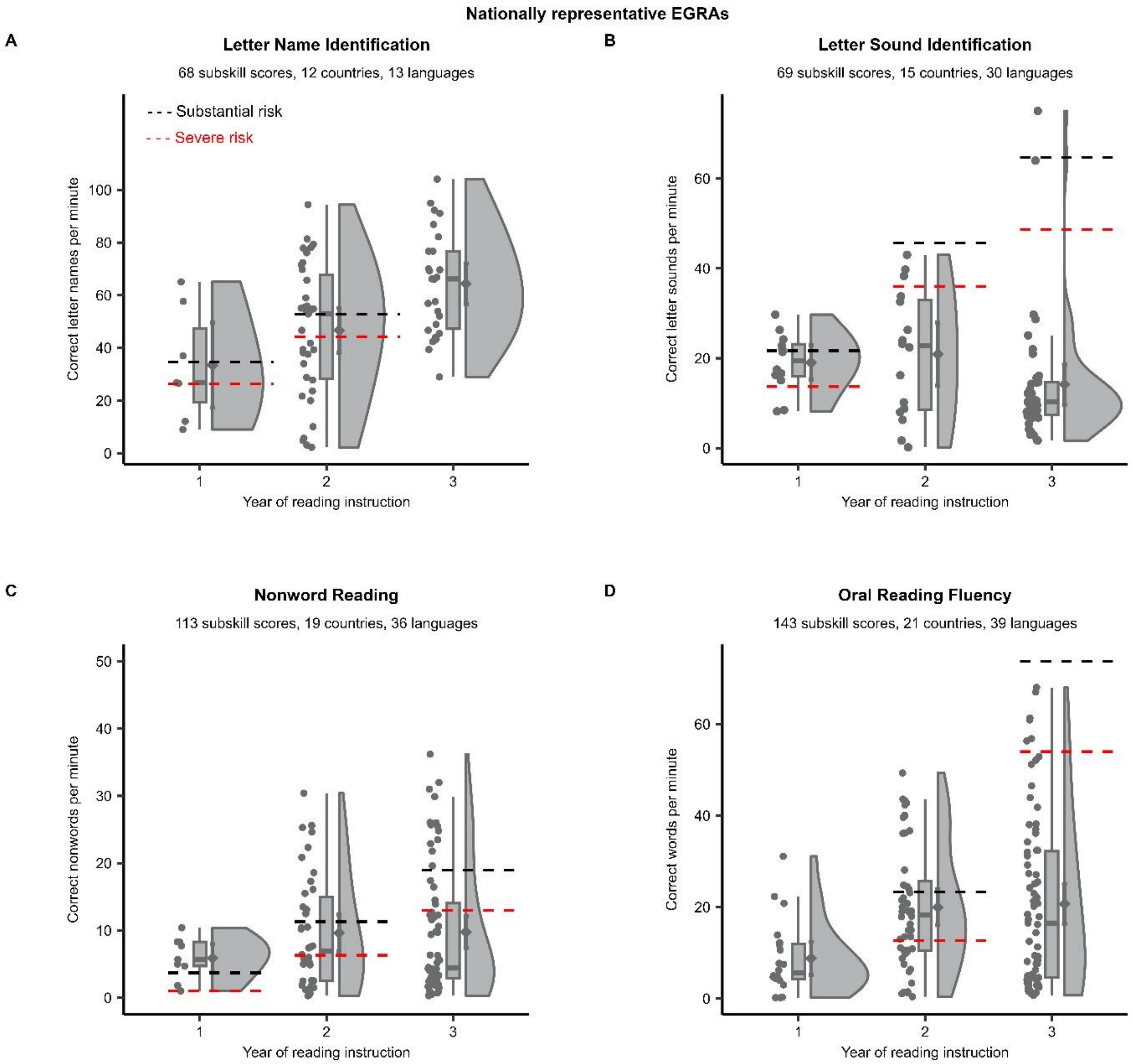

Figure S1. Performance on four reading subskill measures for the first three instructional years in EGRA surveys with nationally-representative samples only. Each panel represents the data from one of the four subskill measures: Panel A - Letter Name Identification, Panel B - Letter Sound Identification, Panel C - Nonword Reading, Panel D - Oral Reading Fluency. For each panel, the number of subskill scores, countries, and languages analyzed is reported under the name of the task. Each panel shows the jittered raw data (the dots representing average subskill score); the four quartiles of the ordered data (the box-and-whiskers plots) with the grey horizontal lines representing the medians (50%), the bounds of the boxes representing the lower and the upper 25% quartiles, and the whiskers representing the expected variation of the data; and the estimated data distribution (the clouds) together with the means (the dots) and the 95% confidence intervals (the error bars). The DIBELS benchmarks (where available) for substantial and severe risk are represented with dashed lines (black and red, respectively) averaged across three time-points (beginning, middle, and end of year).

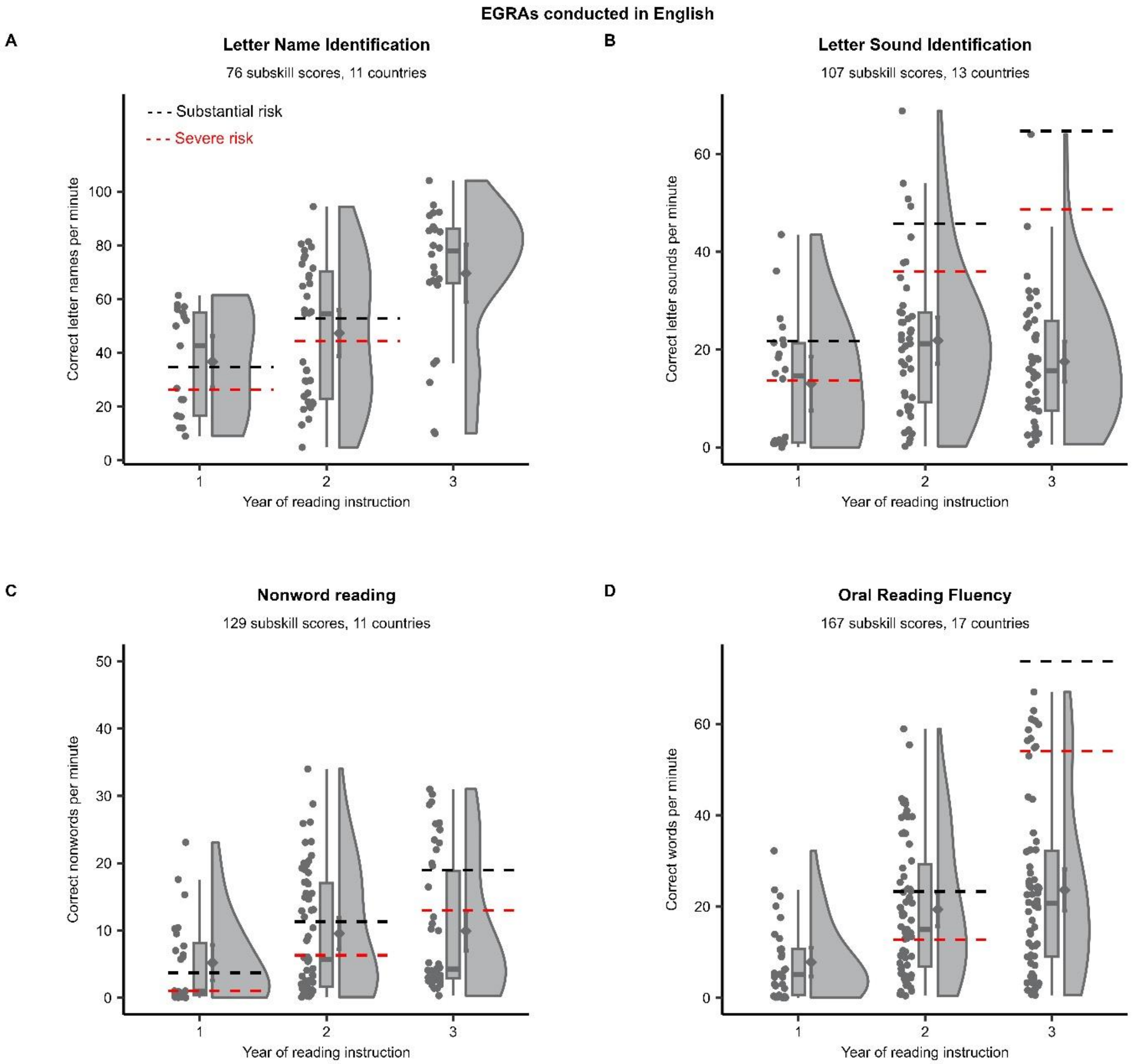

Figure S2. Performance on four reading subskill measures for the first three instructional years in EGRA surveys testing pupils learning to read in English. Each panel represents the data from one of the four subskill measures: Panel A - Letter Name Identification, Panel B - Letter Sound Identification, Panel C - Nonword Reading, Panel D - Oral Reading Fluency. For each panel, the number of subskill scores and countries analyzed is reported under the name of the task. Each panel shows the jittered raw data (the dots representing average subskill score); the four quartiles of the ordered data (the box-and-whiskers plots) with the grey horizontal lines representing the medians (50%), the bounds of the boxes representing the lower and the upper 25% quartiles, and the whiskers representing the expected variation of the data; and the estimated data distribution (the clouds) together with the means (the dots) and the 95% confidence intervals (the error bars). The DIBELS benchmarks (where available) for substantial and severe risk are represented with dashed lines (black and red, respectively) averaged across three time-points (beginning, middle, and end of year).

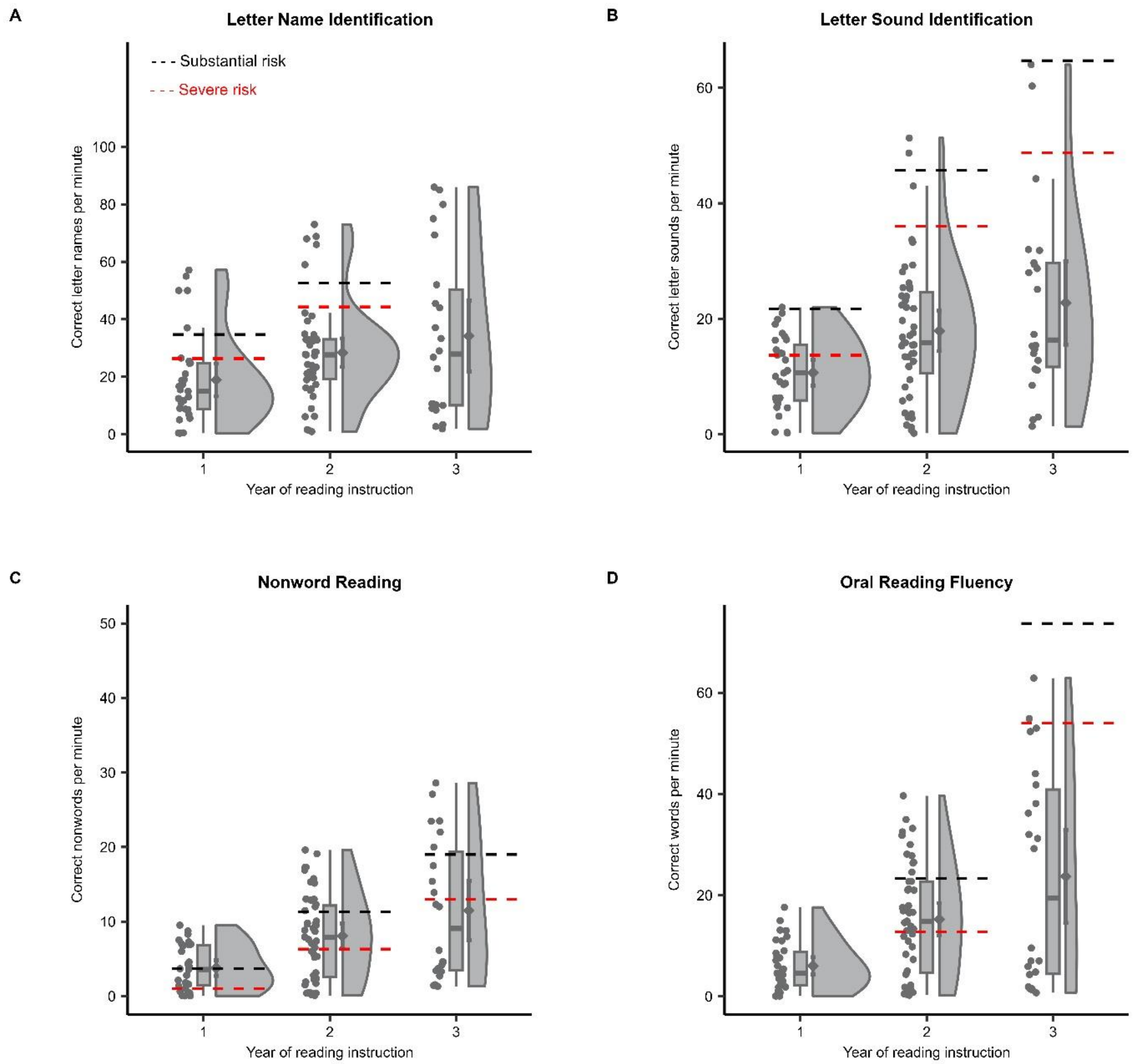

Figure S3. Subskill scores by task and instructional year only for those EGRA surveys that included all four tasks. Each panel represents the data from one of the four decoding tasks: Panel A - Letter Name Identification, Panel B - Letter Sound Identification, Panel C - Nonword Reading, Panel D - Oral Reading Fluency. For each task, these EGRA surveys represent 100 subskill scores from 15 countries and 12 languages. Each panel shows the jittered raw data (the dots representing average subskill score); the four quartiles of the ordered data (the box-and-whiskers plots) with the grey horizontal lines representing the medians (50%), the bounds of the boxes representing the lower and the upper 25% quartiles, and the whiskers representing the expected variation of the data; and the estimated data distribution (the clouds) together with the means (the dots) and the 95% confidence intervals (the error bars). The DIBELS benchmarks (where available) for substantial and severe risk are represented with dashed lines (black and red, respectively) averaged across three time-points (beginning, middle, and end of year).

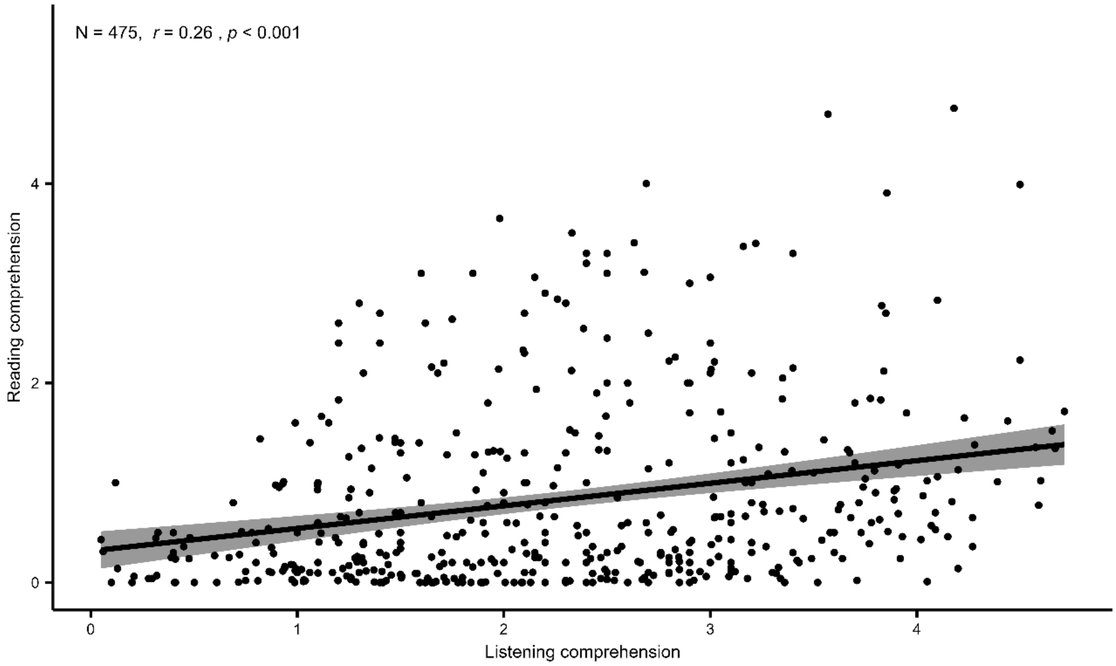

Figure S4. Relationship between performance on the reading comprehension task (on the y-axis) and the listening comprehension task (on the x-axis). The two-sided Pearson correlation coefficient (*r*), the number of data points (*N*) included in the computation of the correlation, and the associated p-value (*p*) are shown in the top left corner. The grey shading around the linear regression lines represents the 95% confidence interval. Scores were included only where a measure from at least one of the decoding tasks under investigation was available (thus ensuring that the samples reflected in this graph overlap with those reflected in Figure 1 in the main text).

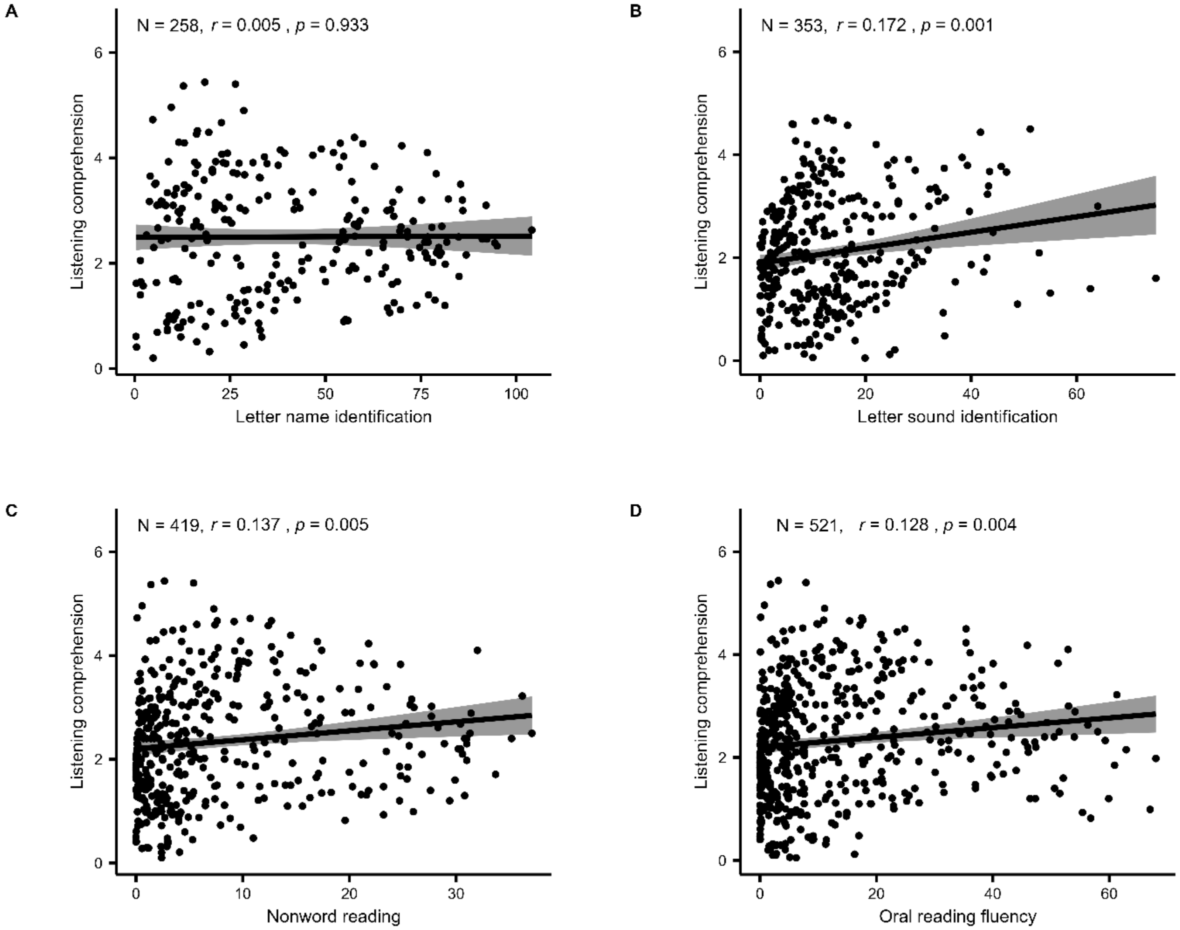

Figure S5. Relationship between performance on the four decoding tasks and on the listening comprehension task. Each panel depicts a scatter plot between the data from the listening comprehension task (on the y-axis) and that from one of the four decoding tasks (on the x-axis): Panel A - Letter Name Identification, Panel B - Letter Sound Identification, Panel C - Nonword Reading, Panel D - Oral Reading Fluency. Two-sided Pearson correlation coefficients (*r*), the number of data points (*N*) included in the computation of the correlations, and the associated p-values (*p*) are shown in the top left corner of each panel. The grey shading around the linear regression lines represents the 95% confidence interval.

Table S1. Languages included in the EGRA database with corresponding EGRA survey numbers. Only languages with an alphabetic writing system were included in the analyses. EGRA survey numbers denoted with an asterisk (*) report aggregated scores across multiple languages.

Language	Writing System	Script	No. of EGRA Surveys	EGRA Survey Number/(s)
French	Alphabetic	Roman/Latin	29	12, 13, 25, 26, 27, 28, 29, 30, 33, 34, 35, 36, 64, 65, 66, 67, 68, 70, 71, 73, 149, 165, 168, 267, 268, 269, 270, 271, 314
Kirundi	Alphabetic	Roman/Latin	2	14, 15
English	Alphabetic	Roman/Latin	75	24, 53, 54, 55, 56, 57, 58, 59, 84, 85, 107, 108, 109, 112, 113, 114, 115, 116, 117, 118, 119, 120, 121, 136, 137, 138, 139, 140, 141, 142, 143, 144, 158, 207, 209, 210, 211, 212, 213, 216, 217, 239, 240, 241, 247, 248, 252, 253, 254, 256, 272, 273, 274, 275, 281, 282, 289, 290, 300, 309, 317, 318, 319, 320, 322, 323, 335, 336, 337, 338, 340, 341, 342, 346, 347
Kiswahili	Alphabetic	Roman/Latin	26	30, 31, 32, 33, 107, 108, 109, 112, 113, 114, 115, 116, 117, 118, 119, 120, 121, 300, 301, 302, 303, 304, 305, 306, 307, 308
Tshiluba	Alphabetic	Roman/Latin	3	30, 32, 33
Lingala	Alphabetic	Roman/Latin	3	30, 32, 33

Spanish	Alphabetic	Roman/Latin	17	45, 60, 61, 62, 63, 74, 75, 169, 170, 197, 198, 199, 242, 243, 244, 245, 246
Dendi	Alphabetic	Roman/Latin	2	46, 47
Afaan Oromo	Alphabetic	Roman/Latin	3	49, 50, 51*
Aff Somali	Alphabetic	Roman/Latin	2	49, 50
Haddiysa	Alphabetic	Roman/Latin	3	49, 50, 52
Sidamu Affo	Alphabetic	Roman/Latin	2	49, 50
Kasem	Alphabetic	Roman/Latin	5	53*, 54*, 55*, 56, 57
Dagaare	Alphabetic	Roman/Latin	5	53*, 54*, 55*, 56, 57
Dagbani	Alphabetic	Roman/Latin	5	53*, 54*, 55*, 56, 57
Nzema	Alphabetic	Roman/Latin	5	53*, 54*, 55*, 56, 57
Asante Twi	Alphabetic	Roman/Latin	5	53*, 54*, 55*, 56, 57
Gonja	Alphabetic	Roman/Latin	5	53*, 54*, 55*, 56, 57
Fante	Alphabetic	Roman/Latin	5	53*, 54*, 55*, 56, 57
Akuapem Twi	Alphabetic	Roman/Latin	7	53*, 54*, 55*, 56, 57, 58, 59
Dangme	Alphabetic	Roman/Latin	5	53*, 54*, 55*, 56, 57
Ga	Alphabetic	Roman/Latin	5	53*, 54*, 55*, 56, 57

Ewe	Alphabetic	Roman/Latin	5	53*, 54*, 55*, 56, 57
Haitian Creole	Alphabetic	Roman/Latin	9	64, 65, 66, 67, 68, 69, 70, 71, 72
Bahasa	Alphabetic	Roman/Latin	6	93, 94, 95, 96, 97, 98
Lubukusu	Alphabetic	Roman/Latin	2	110, 111
Kikamba	Alphabetic	Roman/Latin	2	110, 111
Kiribati	Alphabetic	Roman/Latin	1	122
Albanian	Alphabetic	Roman/Latin	2	145, 146
Malagasy	Alphabetic	Roman/Latin	3	147, 148, 149
Chichewa	Alphabetic	Roman/Latin	12	150, 151, 152, 153, 154, 155, 156, 157, 158, 159, 160, 161
Bamanankan	Alphabetic	Roman/Latin	6	162*, 163*, 164*, 165, 166, 167
Bomu	Alphabetic	Roman/Latin	3	162*, 163*, 164*
Fulfulde	Alphabetic	Roman/Latin	8	162*, 163*, 164*, 200*, 201*, 202, 203, 204
Songhai	Alphabetic	Roman/Latin	3	162*, 163*, 164*
Xichangana	Alphabetic	Roman/Latin	3	181, 182*, 184*
Xirhonga	Alphabetic	Roman/Latin	3	181, 182*, 184*
Echuwabo	Alphabetic	Roman/Latin	2	185, 186

Elomwe	Alphabetic	Roman/Latin	2	185, 186
Emakhuwa	Alphabetic	Roman/Latin	2	185, 186
Hausa	Alphabetic	Roman/Latin	12	200, 201, 202, 203, 204, 205, 206, 207, 208, 209, 212, 213
Zarma	Alphabetic	Roman/Latin	5	200, 201, 202, 203, 204
Tamasheq	Alphabetic	Roman/Latin	2	200*, 201*
Kuanua	Alphabetic	Roman/Latin	1	238
Filipino	Alphabetic	Roman/Latin	8	247, 248, 251, 252, 253, 254, 255, 256
Ilokano	Alphabetic	Roman/Latin	5	247, 249, 250, 252*, 253*
Cebuano	Alphabetic	Roman/Latin	5	249, 250, 251*, 252*, 253*
Hiligaynon	Alphabetic	Roman/Latin	3	249, 250, 251*
Maguindanaoan	Alphabetic	Roman/Latin	2	249, 250
Central Bikol	Alphabetic	Roman/Latin	1	251*
Tagalog	Alphabetic	Roman/Latin	1	251*
Kinyarwanda	Alphabetic	Roman/Latin	9	257, 258, 259, 260, 261, 262, 263, 264, 265
Samoan	Alphabetic	Roman/Latin	1	266

Wolof	Alphabetic	Roman/Latin	4	267, 268, 269, 270
Pulaar	Alphabetic	Roman/Latin	3	267, 268, 269
Seereer	Alphabetic	Roman/Latin	3	267, 268, 269
Setswana	Alphabetic	Roman/Latin	6	276*, 277*, 278, 279, 280, 281
isiXhosa	Alphabetic	Roman/Latin	1	282*
isiZulu	Alphabetic	Roman/Latin	3	276*, 277*, 282*
Sepedi	Alphabetic	Roman/Latin	5	276*, 277*, 283, 284, 285
Xitsonga	Alphabetic	Roman/Latin	3	286, 287, 288
Bari	Alphabetic	Roman/Latin	1	289*
Dinka	Alphabetic	Roman/Latin	1	289*
Ŋatoposa	Alphabetic	Roman/Latin	1	289*
Nuer	Alphabetic	Roman/Latin	1	289*
Zande	Alphabetic	Roman/Latin	1	289*
Portuguese	Alphabetic	Roman/Latin	11	176, 177, 178, 179, 180, 181, 182, 183, 184, 185, 310
Tetum	Alphabetic	Roman/Latin	4	310, 311, 312, 313
Tongan	Alphabetic	Roman/Latin	1	315

Tuvaluan	Alphabetic	Roman/Latin	1	316
Luganda	Alphabetic	Roman/Latin	3	317, 318, 319
Runyankore/Rukiga	Alphabetic	Roman/Latin	3	317, 318, 319
Ateso	Alphabetic	Roman/Latin	1	319
Leblango	Alphabetic	Roman/Latin	1	319
Leb Acöli	Alphabetic	Roman/Latin	2	320, 321
Lugbarati	Alphabetic	Roman/Latin	2	320, 321
Lumasaaba	Alphabetic	Roman/Latin	2	320, 321
Runyoro-Rutooro	Alphabetic	Roman/Latin	2	320, 321
Lhukonzo	Alphabetic	Roman/Latin	2	321, 322
Lugwere	Alphabetic	Roman/Latin	2	321, 322
Lusoga	Alphabetic	Roman/Latin	2	321, 322
Ngakarimojong	Alphabetic	Roman/Latin	2	321, 322
Vietnamese	Alphabetic	Roman/Latin	2	324, 325
Chewa	Alphabetic	Roman/Latin	2	333, 334
Chitonga	Alphabetic	Roman/Latin	5	335, 336, 337, 338, 342

Cinyanja	Alphabetic	Roman/Latin	11	335, 336, 337, 338, 339*, 340*, 341*, 342, 343, 344, 345
Icibemba	Alphabetic	Roman/Latin	8	335, 336, 337, 338, 339*, 340*, 341*, 342
Kiikaonde	Alphabetic	Roman/Latin	5	338, 339*, 340*, 341*, 342
Lunda	Alphabetic	Roman/Latin	2	338, 342
Luvale	Alphabetic	Roman/Latin	2	338, 342
Silozi	Alphabetic	Roman/Latin	5	338, 339*, 340*, 341*, 342
Shona	Alphabetic	Roman/Latin	2	346, 347
Macedonian	Alphabetic	Cyrillic	2	145, 146
Kyrgyz	Alphabetic	Cyrillic	4	123, 124, 125, 126
Russian	Alphabetic	Cyrillic	8	123, 124, 125, 126, 296, 297, 298, 299
Tajik	Alphabetic	Cyrillic	4	296, 297, 298, 299
Dari	Abjad	Arabic	3	1, 2, 3
Pashto	Abjad	Arabic	6	1, 2, 214, 215, 236, 237
Arabic	Abjad	Arabic	32	37, 38, 39, 40, 41, 42, 43, 44, 99, 100, 101, 102, 103, 104, 105, 106, 133, 134, 135, 168, 171, 172, 173, 174, 175, 326, 327, 328, 329, 330, 331, 332

Kanuri	Abjad	Arabic	5	200, 201, 202, 203, 204
Urdu	Abjad	Arabic (Nastaliq variant)	22	214, 215, 218, 219, 220, 221, 222, 223, 224, 225, 226, 227, 228, 229, 230, 231, 232, 233, 234, 235, 236, 237
Sindhi	Abjad	Arabic	3	218, 219, 234
Bangla	Alphasyllabary	Bangla	8	4, 5, 6, 7, 8, 9, 10, 11
Khmer	Alphasyllabary	Khmer	8	16, 17, 18, 19, 20, 21, 22, 23
Tigrigna	Alphasyllabary	Ethiopic	3	48, 49, 50
Amharic	Alphasyllabary	Ethiopic	3	49, 50, 51*
Wolayttatto	Alphasyllabary	Ethiopic	3	49, 50, 52
Hindi	Alphasyllabary	Devanagari	15	76, 77, 78, 79, 80, 81, 82, 83, 86, 87, 88, 89, 90, 91, 92
Kannada	Alphasyllabary	Kannada	2	76, 77
Marathi	Alphasyllabary	Devanagari	2	76, 77
Oriya	Alphasyllabary	Oriya	2	82, 83
Laotian	Alphasyllabary	Lao	6	127, 128, 129, 130, 131, 132
Nepali	Alphasyllabary	Devanagari	10	187, 188, 189, 190, 191, 192, 193, 194, 195, 196
Sinhala	Alphasyllabary	Sinhala	3	291, 292, 293

Tamil	Alphasyllabary	Tamil	2	294, 295

Table S2. Countries included in the EGRA database with corresponding EGRA survey numbers. Note that only data referring to alphabetic writing systems were included in the analyses.

Country	Writing System/(s)	No. of EGRA Surveys	EGRA Survey Number/(s)
Benin	Alphabetic	2	12, 13
Burundi	Alphabetic	2	14, 15
Cameroon	Alphabetic	3	24, 25, 26
Democratic Republic of the Congo	Alphabetic	7	27, 28, 29, 30, 31, 32, 33
Djibouti	Alphabetic	3	34, 35, 36
El Salvador	Alphabetic	1	45
Ghana	Alphabetic	7	53, 54, 55, 56, 57, 58, 59
Guatemala	Alphabetic	4	60, 61, 62, 63
Haiti	Alphabetic	10	64, 65, 66, 67, 68, 69, 70, 71, 72, 73
Honduras	Alphabetic	2	74, 75
Indonesia	Alphabetic	6	93, 94, 95, 96, 97, 98
Kenya	Alphabetic	15	107, 108, 109, 110, 111, 112, 113, 114, 115, 116, 117, 118, 119, 120, 121
Kiribati	Alphabetic	1	122

Kyrgyz Republic	Alphabetic	4	123, 124, 125, 126
Liberia	Alphabetic	9	136, 137, 138, 139, 140, 141, 142, 143, 144
Macedonia	Alphabetic	2	145, 146
Madagascar	Alphabetic	3	147, 148, 149
Malawi	Alphabetic	12	150, 151, 152, 153, 154, 155, 156, 157, 158, 159, 160, 161
Mali	Alphabetic	6	162, 163, 164, 165, 166, 167
Mexico	Alphabetic	2	169, 170
Mozambique	Alphabetic	11	176, 177, 178, 179, 180, 181, 182, 183, 184, 185, 186
Nicaragua	Alphabetic	3	197, 198, 199
Nigeria	Alphabetic	9	205, 206, 207, 208, 209, 210, 211, 212, 213
Papua New Guinea	Alphabetic	4	238, 239, 240, 241
Peru	Alphabetic	5	242, 243, 244, 245, 246
Philippines	Alphabetic	10	247, 248, 249, 250, 251, 252, 253, 254, 255, 256
Rwanda	Alphabetic	9	257, 258, 259, 260, 261, 262, 263, 264, 265
Samoa	Alphabetic	1	266

Senegal	Alphabetic	5	267, 268, 269, 270, 271
Sierra Leone	Alphabetic	4	272, 273, 274, 275
South Africa	Alphabetic	13	276, 277, 278, 279, 280, 281, 282, 283, 284, 285, 286, 287, 288
South Sudan	Alphabetic	2	289, 290
Tajikistan	Alphabetic	4	296, 297, 298, 299
Tanzania	Alphabetic	9	300, 301, 302, 303, 304, 305, 306, 307, 308
The Gambia	Alphabetic	1	309
Timor-Leste	Alphabetic	4	310, 311, 312, 313
Togo	Alphabetic	1	314
Tonga	Alphabetic	1	315
Tuvalu	Alphabetic	1	316
Uganda	Alphabetic	6	317, 318, 319, 320, 321, 322
Vanuatu	Alphabetic	1	323
Vietnam	Alphabetic	2	324, 325
Zambia	Alphabetic	13	333, 334, 335, 336, 337, 338, 339, 340, 341, 342, 343, 344, 345
Zimbabwe	Alphabetic	2	346, 347

Niger	Alphabetic & Abjad	5	200, 201, 202, 203, 204
Mauritania	Alphabetic & Abjad	1	168
Ethiopia	Alphabetic & Alphasyllabary	7	46, 47, 48, 49, 50, 51, 52
India	Alphabetic & Alphasyllabary	17	76, 77, 78, 79, 80, 81, 82, 83, 84, 85, 86, 87, 88, 89, 90, 91, 92
Pakistan	Alphabetic & Abjad	24	214, 215, 216, 217, 218, 219, 220, 221, 222, 223, 224, 225, 226, 227, 228, 229, 230, 231, 232, 233, 234, 235, 236, 237
Afghanistan	Abjad	3	1, 2, 3
Egypt	Abjad	8	37, 38, 39, 40, 41, 42, 43, 44
Iraq	Abjad	1	99
Jordan	Abjad	7	100, 101, 102, 103, 104, 105, 106
Lebanon	Abjad	3	133, 134, 135
Morocco	Abjad	5	171, 172, 173, 174, 175
West Bank	Abjad	2	326, 327
Yemen	Abjad	5	328, 329, 330, 331, 332
Bangladesh	Alphasyllabary	8	4, 5, 6, 7, 8, 9, 10, 11

Cambodia	Alphasyllabary	8	16, 17, 18, 19, 20, 21, 22, 23
Lao PDR	Alphasyllabary	6	127, 128, 129, 130, 131, 132
Nepal	Alphasyllabary	10	187, 188, 189, 190, 191, 192, 193, 194, 195, 196
Sri Lanka	Alphasyllabary	5	291, 292, 293, 294, 295

Table S3. Percentage of subskill scores falling below the 'substantial risk' and 'severe risk' DIBELS benchmarks [9] in each instructional year for nationally-representative samples only.

Risk Level<br>Instructional Year	Letter Name Identification	Letter Sound Identification	Nonword Reading	Oral Reading Fluency
Substantial Risk<br>Year 1	57%	58%	22%	No Benchmark
Substantial Risk<br>Year 2	49%	100%	66%	69%
Substantial Risk<br>Year 3	No Benchmark	98%	80%	100%
Severe Risk<br>Year 1	29%	17%	0%	No Benchmark
Severe Risk<br>Year 2	46%	81%	47%	33%
Severe Risk<br>Year 3	No Benchmark	95%	73%	92%

Table S4. Percentage of subskill scores falling below the 'substantial risk' and 'severe risk' DIBELS benchmarks [9] in each instructional year. Scores from EGRA surveys testing pupils learning to read in English only.

Risk Level<br>Instructional Year	Letter Name Identification	Letter Sound Identification	Nonword Reading	Oral Reading Fluency
Substantial Risk<br>Year 1	47%	77%	54%	No Benchmark
Substantial Risk<br>Year 2	49%	91%	59%	66%
Substantial Risk<br>Year 3	No Benchmark	100%	74%	100%
Severe Risk<br>Year 1	41%	45%	32%	No Benchmark
Severe Risk<br>Year 2	49%	84%	14%	37%
Severe Risk<br>Year 3	No Benchmark	98%	43%	85%

Table S5. Percentage of subskill scores falling below the 'substantial risk' and 'severe risk' benchmarks [9] in each instructional year in those EGRA surveys (*N* = 27) that included all four decoding measures (100 subskill scores per task).

Risk Level Instructional Year	Letter Name Identification	Letter Sound Identification	Nonword Reading	Oral Reading Fluency
Substantial Risk Year 1	84%	97%	55%	No Benchmark
Substantial Risk Year 2	89%	96%	70%	77%
Substantial Risk Year 3	No Benchmark	100%	73%	100%
Severe Risk Year 1	89%	61%	19%	No Benchmark
Severe Risk Year 2	89%	94%	38%	40%
Severe Risk Year 3	No Benchmark	91%	59%	91%

Table S6. Number of EGRA surveys, sub-surveys, and subskill scores for tasks other than the four tasks discussed in the main text. We also report the number of countries these surveys were conducted in and the number of languages these surveys assessed. This table includes data from alphabetic writing systems only, and only from those EGRA reports from which information about the *number* of correct responses could be extracted.

Task	Number of EGRA surveys	Number of EGRA sub-surveys	Number of subskill scores	Number of countries	Number of languages
Listening Comprehension	147	468	598	39	83
Reading Comprehension	206	601	782	47	92
Oral Vocabulary	51	137	195	14	17
Initial Sound Identification	73	215	278	29	35
Phoneme Segmentation	10	33	49	2	9
Syllable Identification	39	80	99	10	19
Familiar Word Reading	145	414	526	41	57
Orientation to print	26	76	97	8	22
Dictation	40	115	137	17	19
Maze (Cloze)	3	9	16	1	2

Note: 19 EGRA sub-surveys, for some tasks, reported *percentages* of correct responses but did not report the total number of questions that were administered. These sub-surveys are 34a, 35a, 36a, 60a, 60b, 61a, 62a, 63a, 63b, 71a, 71c, 71d, 122a, 122b, 149a, 149b, 238a, 270a, 270b, 270c, 270d, 270e,

277a, 309a, 309b, 309c, 311a, 311b, 311c, 315a, 315b, 315c, 316a, 316b, 316c; and the affected tasks include listening comprehension, reading comprehension, initial sound identification, phoneme segmentation, oral vocabulary, and dictation. Because the numbers of correct responses for these tasks and sub-surveys could not be estimated, we did not include them in this table.

Table S7. Pearson's skewness coefficients for all dependent variables used in the analysis.

Task	Instructional year	Pearson's skewness coefficient
Letter name identification	1	1.13
	2	0.86
	3	−0.12
Letter sound identification	1	1.25
	2	1.23
	3	1.59
Nonword reading	1	1.96
	2	1.03
	3	0.45
Oral reading fluency	1	2.05
	2	1.21
	3	0.80

Supplementary Appendix

The analysis reported here was performed to ensure that the results of the main analysis were not biased by the fact that the data were modelled using the student t-distribution. While the student t-distribution can account for heavy tails (i.e., greater chance of extreme values compared to the normal distribution), it is symmetric. Yet, a visual inspection of the data suggests that they could have been generated by a skewed process. Thus, in this analysis, we defined the likelihood using the skewed t-distribution (Hansen et al., 1994). The code for this analysis is available on the OSF page of this project (https://osf.io/6s23f/).

Method

The skewed t-distribution is a special case of the skewed generalised t-distribution (Hansen et al., 2010; Theodossiou, 1998). The probability density function of the skewed generalised t-distribution is defined as follows:

$$f_{SGT}(x;\ \mu, \sigma, \lambda, p, q) = \frac{p}{2\nu\sigma q^{\frac{1}{p}} \mathrm{B}\left(\frac{1}{p}, q\right) \left[1 + \frac{|x - \mu + m|^{p}}{q(\nu\sigma)^{p}\left(1 + \lambda\, sgn(x - \mu + m)\right)^{p}}\right]^{\frac{1}{p}+q}}$$

where μ is the location parameter, σ is the scale parameter, λ is the skewness parameter, p and q are parameters that control the kurtosis of the distribution, and m and ν are functions that are used to scale the distribution to appropriately match its parametrisations (see Theodossiou, 1998, for further information). In the skewed generalised t-distribution, parameters σ, λ, p, q have the following restrictions:

$$\{(\sigma, \lambda, p, q): \sigma > 0, -1 < \lambda < 1, p > 0, q > 0\}$$

In the skewed t-distribution, the restrictions on the σ, λ, q parameters are unchanged, and the p parameter has a fixed value of 2, such that the probability density function of this distribution is as follows:

$$f_{SGT}(x;\ \mu, \sigma, \lambda, p = 2, q) =$$

$$f_{SGT}(x;\ \mu, \sigma, \lambda, q) =$$

$$= \frac{\Gamma(\frac{1}{2} + q)}{\nu\sigma(\pi q)^{\frac{1}{2}}\,\Gamma(q)\left[1 + \frac{|x - \mu + m|^2}{q(\nu\sigma)^2\left(1 + \lambda\, sgn(x - \mu + m)\right)^2}\right]^{\frac{1}{2}+q}}$$

where

$$m = \lambda\nu\sigma\,\frac{2q^{\frac{1}{2}}\,\Gamma\left(q - \frac{1}{2}\right)}{\pi^{\frac{1}{2}}\,\Gamma(q)}$$

and

$$\nu = \frac{1}{q^{\frac{1}{2}}\sqrt{(1 + 3\lambda^2)\frac{1}{2q - 2} - \frac{4\lambda^2}{\pi}\left(\frac{\Gamma(q - \frac{1}{2})}{\Gamma(q)}\right)^2}}$$

The model was specified in Stan, a probabilistic programming language for statistical modelling (Stan Development Team, 2024). In Stan, the models are fit using two Markov chain Monte Carlo (MCMC) algorithms, an advanced dynamic Hamiltonian Monte Carlo algorithm (Betancourt, 2016; Betancourt & Girolami, 2013) and its adaptive variant, the No-U-Turn sampler (NUTS; Hoffman & Gelman, 2014), which is thought to be more efficient than the Gibbs sampler used in other probabilistic languages (e.g., (Win)BUGS; Lunn et al., 2000; JAGS; Plummer, 2003). Stan can be accessed through several interfaces (e.g., R, Python); in this analysis, we used the R package *rstan* (Guo et al., 2019), which integrates Stan with R (R Core Team, 2023). One advantage of Stan is that, when a required distribution is not available, it can be custom built using the functions available in the Stan language. Because the skewed t-distribution is not included in Stan, we defined it using the functions provided in the *Helpful Stan functions* project (https://spinkney.github.io/helpful_stan_functions/index.html).

To facilitate comparison with the main analysis (where the data is modelled with a t-distribution), the priors for the μ and σ parameters were identical to those reported in the main body of this article. In Analysis 1 (comparing performance across the instructional years), the μ parameter was defined using the normal distribution, with the mean set to the mean of the pooled data y, and the standard deviation set to 1,000 times the standard deviation of the pooled data y, $\mu \sim Normal\big(mean(y), 1000 \times sd(y)\big)$. The prior on the standard deviation was defined as a uniform distribution ranging from one thousandth of the standard deviation of the pooled data to one thousand times the standard deviation of the pooled data, $\sigma \sim Uniform\left(\frac{sd(y)}{1000}, sd(y) \times 1000\right)$. In Analysis 2 (comparing performance against the benchmarks), the priors were defined based on the performance expected for a given task and instructional year within the DIBELS framework in order for the pupils to not fall into the substantial risk category (see main body of the paper for further detail).

The priors on the λ and q parameters were identical in both analyses. For λ, we used the normal distribution with the mean 0 and the standard deviation of 0.5, to allow for both positive and negative skew, and, for q, we used the gamma distribution with the shape parameter (α) set to 2 and the inverse scale parameter (β) set to $\frac{1}{0.1} = 10$. The effect sizes were computed using the formulae provided in the main body of the article.

Results

The results of this analysis for the parameters of interest[1] are reported in Table SA1 (Analysis 1) and Table SA2 (Analysis 2). The new models take into account that the data may have been generated by a skewed process, and, consequently, the new estimates differ slightly from those provided by the original analysis. In some cases (e.g., performance on the nonword reading task in Year 1), the new models were able to

[1] The estimates for all other parameters are available in the .html file on the OSF page of this project (https://osf.io/6s23f/).

recover the mean and the standard deviation better than the original models; however, for all parameters, the two analyses produced very similar 95% credibility intervals.

Overall, the results of the new analysis mirror those reported in the main body of the article: in all tasks except for the letter sound identification, the subskill scores improved with each instructional year (see Table SA1). For the letter sound identification task, the subskill scores in Year 2 were superior to those in Year 1; however, there was no difference between the scores in Year 2 and Year 3. Despite this improvement in scores across the instructional years, for each year and task, the average performance was lower than that expected under the 'substantial risk' benchmark (see Table SA2). For all tasks except for the nonword reading task, the absolute values of the effect size estimates increase as a function of instructional year, suggesting that, on average, the performance gap between the pupils' actual performance and the performance expected under the 'substantial risk' benchmark widens with each instructional year.

Table SA1. Differences in pupils' performance on each of the four decoding tasks across the three instructional years. For each parameter, we report the mean point estimate (top row in the results columns) and the 95% HDI credibility interval (bottom row) of the posterior distribution (i.e., a range of credible values for the effect of interest). The effect size is Cohen's d (Cohen, 1988) computed from the posterior distribution, with the mean representing the mean standardized distance between pupils' performance across the instructional years and the 95% HDI representing the uncertainty around this point estimate. Estimates for the means and SDs reflect the number of correct items (letter names, letter sounds, nonwords, words) per minute.

Task	Contrast	Mean point estimate and the 95% HDI of the posterior distribution		
		Difference in means	Difference in standard deviations	Effect size
Letter name identification	Year 2 vs. Year 1	7.08<br>[1.70, 12.19]	5.48<br>[1.24, 9.54]	0.34<br>[0.08, 0.56]
	Year 3 vs. Year 2	17.93<br>[11.28, 24.89]	12.00<br>[6.84, 17.33]	0.65<br>[0.42, 0.86]
Letter sound identification	Year 2 vs. Year 1	6.52<br>[4.13, 8.94]	5.03<br>[2.88, 7.73]	0.50<br>[0.33, 0.66]
	Year 3 vs. Year 2	−0.81<br>[−3.38, 1.72]	−1.49<br>[−3.75, 0.72]	−0.06<br>[−0.24, 0.13]
Nonword reading	Year 2 vs. Year 1	5.15<br>[4.04, 6.40]	5.61<br>[4.13, 7.27]	0.64<br>[0.52, 0.77]
	Year 3 vs. Year 2	5.87<br>[4.23, 7.62]	4.12<br>[2.82, 5.57]	0.68<br>[0.50, 0.84]
Oral reading fluency	Year 2 vs. Year 1	8.37<br>[6.97, 9.82]	9.52<br>[7.59, 11.44]	0.67<br>[0.58, 0.76]
	Year 3 vs. Year 2	10.52<br>[8.18, 13.21]	8.82<br>[6.24, 12.28]	0.66<br>[0.52, 0.78]

Table SA2. Performance on each task compared to that expected under the substantial risk benchmark for each instructional year (prior distribution). For each parameter, the prior was defined using the Normal distribution (mean and SD are indicated in parentheses), and the results columns show the mean point estimate (top row) and the 95% HDI credibility interval (bottom row) of the posterior distribution. The effect size is Cohen's d (Cohen, 1988) computed from the posterior distribution, with the mean representing the mean standardized distance between performance expected under the 'substantial risk' benchmark and that achieved by the pupils, and the 95% HDI describing the uncertainty associated with this mean point estimate. The substantial risk benchmark for each task and instructional year is reported in the rightmost column, with the top row displaying the cut-off scores for the beginning, middle, and end of each year, and the bottom row showing the average score across these three time points. Estimates for the means reflect the number of correct items (letter names, letter sounds, nonwords, words) per minute.

Task	Instructional year	Prior	Mean point estimate and the 95% HDI of the posterior distribution		'Substantial risk' benchmarks
Letter name identification	1	$Normal(35,5)$	24.20<br>[20.52, 28.28]	−0.61<br>[−0.95, −0.30]	25, 37, 42<br>*M* = 34.7
	2	$Normal(53,5)$	32.18<br>[28.82, 35.91]	−0.85<br>[−1.10, −0.62]	42, 57, 59<br>*M* = 52.7
Letter sound identification	1	$Normal(20,7)$	11.09<br>[9.55, 12.79]	−1.04<br>[−1.39, −0.70]	9, 25, 31<br>*M* = 21.7
	2	$Normal(45,7)$	18.16<br>[16.56, 19.97]	−1.96<br>[−2.28, −1.64]	30, 52, 55<br>*M* = 45.7
	3	$Normal(60,8)$	17.53<br>[15.45, 19.82]	−3.53<br>[−4.25, −2.56]	50, 68, 76<br>*M* = 64.7
Nonword reading	1	$Normal(3,1.5)$	3.09<br>[2.53, 3.71]	−0.18<br>[−0.40, 0.00]	1, 3, 7<br>*M* = 3.7
	2	$Normal(10,5)$	8.42<br>[7.57, 9.28]	−0.40<br>[−0.55, −0.25]	5, 14, 15<br>*M* = 11.3
	3	$Normal(18,0.7)$	16.53<br>[15.38, 17.69]	−0.20<br>[−0.32, −0.10]	15, 20, 22<br>*M* = 19
Oral reading fluency	2	$Normal(23,8)$	12.71<br>[11.46, 14.07]	−0.80<br>[−1.04, −0.59]	10, 21, 39<br>*M* = 23.3
	3	$Normal(73,13)$	23.71<br>[21.85, 25.69]	−2.77<br>[−3.11, −2.43]	49, 78, 94<br>*M* = 73.7

References

Betancourt, M. J. (2016). *Identifying the optimal integration time in Hamiltonian Monte Carlo*. http://arxiv.org/abs/1601.00225.

Betancourt, M., & Girolami, M. (2013). *Hamiltonian Monte Carlo for hierarchical models*. http://arxiv.org/abs/1312.0906.

Cohen, J. (1988). *Statistical power analysis for the behavioral sciences* (2nd ed.). Hillsdale, NJ: Erlbaum.

Guo, J., Gabry, J., & Goodrich, B. (2019). *rstan: R Interface to Stan*. https://CRAN.R-project.org/package=rstan.

Hansen, B. E. (1994). Autoregressive conditional density estimation. *International Economic Review*, 35 (3), 705–730. https://doi.org/10.2307/2527081

Hansen, C., McDonald, J., & Newey, W. (2010). Instrumental variables estimation with flexible distributions. *Journal of Business and Economic Statistics*, 28, 13–25. https://doi.org/10.1198/jbes.2009.06161

Hoffman, M. D., & Gelman, A. (2014). The No-U-Turn Sampler: adaptively setting path lengths in Hamiltonian Monte Carlo. *Journal of Machine Learning Research*, 15 (1): 1593–1623. http://dl.acm.org/citation.cfm?id=2627435.2638586

Lunn, D. J., Thomas, A., Best, N.G., & Spiegelhalter, D.J. (2000). WinBUGS - A Bayesian modelling framework: Concepts, structure, and extensibility. *Statistics and Computing*, 10 (4): 325–337. https://doi.org/10.1023/A:1008929526011

Plummer, M. (2003). JAGS: A program for analysis of Bayesian graphical models using Gibbs sampling. *Proceedings of the 3rd International Workshop on Distributed Statistical Computing* (DSC 2003), March 20-22, Vienna, Austria. ISSN 1609-395X

R Core Team (2023). *R: A Language and Environment for Statistical Computing*. Vienna, Austria: R Foundation for Statistical Computing. https://www.R-project.org/

Stan Development Team (2024). *Stan Modeling Language Users Guide and Reference Manual*, 2.35. https://mc-stan.org

Theodossiou, P. (1998). Financial data and the Skewed Generalized T distribution. *Management Science*, 44 (12–part–1), 1650–1661. https://doi.org/10.1287/mnsc.44.12.1650
